# Inactivity and Sitting Time in Later Life: A Saudi National Profile and Policy Implications for Vision 2030

**DOI:** 10.3390/medicina61122095

**Published:** 2025-11-25

**Authors:** Saad M. Bindawas

**Affiliations:** Department of Rehabilitation Sciences, College of Applied Medical Sciences, King Saud University, P.O. Box 10219, Riyadh 11433, Saudi Arabia; sbindawas@ksu.edu.sa

**Keywords:** older adults, physical inactivity, sedentary behavior, Saudi Arabia, sex differences

## Abstract

*Background and Objectives:* Saudi Arabia’s aging population faces significant challenges from physical inactivity and prolonged sedentary behavior. This study quantified the prevalence of physical inactivity and sedentary behavior among older adults (aged 65 years and above) compared with those aged 55–64 years. *Materials and Methods:* We conducted a secondary analysis of data from the 2013 Saudi Health Interview Survey. The analysis focused on adults aged 55–64 years and those aged 65 years and older, stratified by sex. Physical inactivity was defined as <150 min of moderate-to-vigorous activity per week, and sedentary behavior as ≥6 h per day sitting or screen time. *Results:* Among adults ≥65 years (n = 875; 517 men, 358 women), 70.6% of women (95% CI 63.4–77.8) and 37.9% of men (95% CI 32.3–43.5) were inactive (difference: 32.7 percentage points). 42.7% of women (95% CI, 34.7–50.7%) and 32.0% of men (95% CI, 26.3–37.7%) reported ≥6 h of daily sitting. From ages 55–64 to ≥65, inactivity increased by 16.4 percentage points in women and 10.7 percentage points in men, while sitting increased by 9.5 percentage points and 8.5 pp, respectively. *Conclusions:* Older Saudi adults, especially women, face significant challenges related to physical inactivity and sedentary lifestyles, particularly after age 65. Addressing these issues through gender-responsive policies and interventions is vital for promoting health as part of Saudi Vision 2030.

## 1. Introduction

Insufficient physical activity and prolonged sedentary time are well-established, independent, and synergistic risk factors for noncommunicable diseases (NCDs), functional decline, cognitive impairment, and premature mortality among older adults [1,2,3]. Meta-analytic evidence indicates that inadequate physical activity increases mortality risk, while extended sedentary behavior contributes additional harm, which is partially mitigated by meeting physical activity guidelines [4,5]. A landmark multi-cohort study involving over 4 million adult-years demonstrated that the mortality benefits of physical activity increase with age, underscoring the importance of promoting movement throughout the lifespan [6].

The World Health Organization (WHO) 2020 guidelines recommend that adults aged 65 years and older engage in 150–300 min per week of moderate-intensity aerobic activity, perform muscle-strengthening exercises twice a week, and minimize sedentary behavior [7]. However, older adults, particularly in the Eastern Mediterranean region, consistently fall short of these targets, especially in low- and middle-income countries [8].

Saudi Arabia is undergoing rapid population aging and urbanization, which increases the public health importance of understanding movement behaviors among older adults [9]. Chronic diseases now account for the majority of the Kingdom’s disease burden. Systematic reviews have documented persistently high levels of physical inactivity across Saudi populations, with pronounced disparities affecting women [10,11]. A 2024 analysis estimated that insufficient physical activity contributes to an annual economic loss of 0.49% to 0.68% of the gross domestic product [12].

Although the 2013 Saudi Health Interview Survey (SHIS) provided nationally representative estimates of physical activity and sedentary behavior among adults, a focused, sex-stratified analysis of older adults (aged 65 years and above) has not been systematically documented in the peer-reviewed literature. Similarly, national-level data on behavioral transitions around the retirement threshold (age 65) remain limited, despite this being a potentially critical juncture for intervention [13,14]. This gap hinders the development of targeted strategies aligned with Saudi Arabia’s health policy priorities.

Saudi Vision 2030 aims to increase physical activity and improve the quality of life among aging citizens [15]. Physical inactivity and sedentary behavior are distinct: inactivity refers to insufficient moderate-to-vigorous physical activity (MVPA), whereas sedentary behavior involves activities (≤1.5 METs) performed in a sitting or reclining posture [16,17,18]. Notably, individuals may meet MVPA guidelines yet accumulate excessive sedentary time—termed the “active couch potato” [16,19]. Evidence suggests that even 30–40 min of daily MVPA partially offsets the health risks of prolonged sitting [4,20].

Recent studies show that behaviors often co-occur with nutritional and metabolic disturbances in older adults [21,22]. A 2025 study found that physically inactive older individuals had elevated oxidative stress markers, reduced antioxidant capacity, and lower serum levels of protective micronutrients (e.g., vitamins E and C, selenium, zinc) [21,22]. A 2023 systematic review further identified biological pathways linking sedentary behavior to increased inflammation and impaired antioxidant defenses. These findings highlight the importance of integrating nutritional considerations into interventions targeting older adults with dual burdens of inactivity and sedentary behavior.

This study addresses a critical data gap by presenting a comprehensive national profile of physical inactivity and sedentary behavior among Saudi adults aged 65 years and above, using publicly available SHIS 2013 data [23]. The primary objectives are:

(1) to estimate the prevalence of physical inactivity (<150 min/week MVPA) and sedentary behavior (≥6 h/day sitting, excluding sleep) among adults ≥ 65 years, with sex-stratified detail and 95% confidence intervals;
(2) to quantify age-related changes in these behaviors from ages 55–64 to ≥65 years, identifying key transition periods; and
(3) to provide evidence to inform gender-responsive prevention strategies and policy development within the framework of Saudi Vision 2030.

## 2. Materials and Methods

### 2.1. Study Design and Data Source

This secondary analysis examined aggregated, design-weighted prevalence estimates from the 2013 Saudi Health Interview Survey (SHIS), a nationally representative, multistage, cross-sectional survey conducted by the Saudi Ministry of Health (MOH) in collaboration with the Institute for Health Metrics and Evaluation (IHME). The complete SHIS 2013 report is publicly accessible via the IHME Global Health Data Exchange [23]: https://www.healthdata.org/sites/default/files/files/Projects/KSA/Saudi-Health-Interview-Survey-Results.pdf (accessed on 25 September 2025).

Data source specification: This analysis utilized exclusively aggregated, design-weighted prevalence estimates and standard errors published in the official SHIS 2013 results report (Tables 13–18 of the printed report), not individual-level microdata. Individual-level records were unavailable to the analyst. Consequently, statistical modeling, multivariable adjustment, and formal hypothesis testing were not possible. All reported differences are descriptive contrasts expressed as percentage point (pp) differences without confidence intervals, as the variance–covariance matrices necessary for calculating CIs around contrasts were not provided in the published report.

As the author of this paper, I would like to disclose that I utilized Grammarly to assist in the editing and polishing stages of my work. This tool helped enhance the clarity and overall quality of the manuscript.

### 2.2. Survey Design and Weighting Methodology

The SHIS employed multistage stratified probability sampling across the Kingdom’s 13 administrative regions. Within each region, enumeration areas served as primary sampling units (PSUs), with 14 households randomly selected per PSU, resulting in approximately 12,000 households being contacted. From each household, one adult respondent aged 15 years or older was randomly selected using DatStat software. The survey achieved a household response rate of 90% (10,827 completed interviews).

All published prevalence estimates incorporated post-stratified design weights. Individual selection weights were calculated as the inverse of the probability of selection and then post-stratified using the ratio of the 2013 Saudi census population percentages to the sample percentages for each age–sex group. All reported estimates reflect these design-weighted, post-stratified calculations and are therefore representative of the Saudi population.

### 2.3. Study Sample and Age-Band Rationale

Published prevalence estimates were extracted for two age bands:

- Late middle age: 55–64 years (unweighted n = 862; 439 men, 423 women)
- Older adults: ≥65 years (unweighted n = 875; 517 men, 358 women)

Age-band rationale: The 55–64 and ≥65 year age bands were selected to frame the retirement transition. In Saudi Arabia, the typical retirement age ranges from 60 to 65 years; the ≥65 category encompasses the population that has established itself in retirement, characterized by accumulated behavioral changes. The contrast between these bands quantifies the magnitude of behavioral shift at this critical life-course juncture.

Each estimate was stratified by sex. All percentages are design-weighted and representative of the Saudi population; unweighted sample sizes are provided in tables to document the raw survey respondent counts.

### 2.4. Data Collection Procedures

Data were collected via Computer-Assisted Personal Interviewing (CAPI) on portable computers programmed in Arabic by IHME in collaboration with MOH. Interviewers received comprehensive, standardized training in all assessment protocols, including behavioral assessment and physical measurement techniques. Critically, each respondent was interviewed by a same-sex interviewer to align with Saudi cultural norms and ensure respondent comfort and response validity. The questionnaire was administered entirely in Arabic, the respondent’s native language.

### 2.5. Measurement of Outcomes

Physical Inactivity: Assessed using IPAQ-adapted constructs. Respondents reported frequency and duration of vigorous-intensity and moderate-intensity activities across occupational, transportation, and leisure domains. Physical inactivity was defined as <150 min/week of moderate-to-vigorous physical activity (MVPA), operationalized as “not active” in the SHIS and aligned with WHO 2020 guidelines and Sedentary Behavior Research Network (SBRN) 2017 consensus definitions [7,24].

General Sedentary Time: Total daily sitting or reclining time (excluding sleep), across all contexts (occupational, home, transportation, leisure). High sedentary behavior was defined as ≥6 h/day, consistent with epidemiological surveillance thresholds.

Screen-Based Sedentary Time: Time spent watching television or using a computer. High screen time was defined as ≥6 h/day. Note: 2013 predates the proliferation of smartphones; screen time primarily reflects television and computer use.

Alignment with international standards: Both physical activity and sedentary behavior measures align with the IPAQ methodology and SBRN consensus terminology [24,25,26]. Important measurement consideration: Self-reported sedentary time via IPAQ typically underestimates actual sedentary duration by approximately 2–4 h per day when compared to device-based, objective measures such as accelerometry, which introduces conservative prevalence estimates.

### 2.6. Statistical Analysis

Analyses were descriptive. All prevalence estimates and standard errors were extracted directly from the published SHIS 2013 tables. For individual estimates, 95% confidence intervals were calculated using the formula: estimate ± 1.96 × standard error. Age-related comparisons were expressed as absolute percentage point (pp) differences. Formal hypothesis testing and confidence interval calculations for between-group differences were not performed because the SHIS public dataset did not provide variance–covariance matrices necessary for calculating CIs around contrasts or conducting multivariable modeling.

Statistical calculations were performed using Microsoft Excel 365 (Microsoft Corporation, Redmond, WA, USA), with a focus on descriptive statistics.

### 2.7. Reporting Standards and Ethical Considerations

This analysis adhered to the Strengthening the Reporting of Observational Studies in Epidemiology (STROBE) checklist for cross-sectional studies. Ethical review and approval were waived because the analysis utilized publicly available, fully anonymized, aggregated data from which individual participants could not be identified [27]. No informed consent was required.

## 3. Results

Analyses were descriptive and based on aggregated, design-weighted prevalence estimates from the 2013 SHIS. We report sex-stratified levels among adults aged 65 years and older, and the descriptive change from ages 55–64 to 65 years and older; 95% confidence intervals (CIs) are shown for single estimates, while CIs for differences are not reported because covariance between age-band estimates is not available in the public tables.

### 3.1. Prevalence Among Older Adults (≥65 Years)

The unweighted sample of individuals aged 65 years and above included 875 respondents (men, n = 517; women, n = 358), representing the Saudi population in this age group after applying design weights.

Regarding physical inactivity (defined as less than 150 min per week of moderate to vigorous physical activity, categorized as “not active” according to the IPAQ), the findings were as follows: Women: 70.6% (95% CI 63.4–77.8), and Men: 37.9% (95% CI 32.4–43.4)

**Figure 1:**
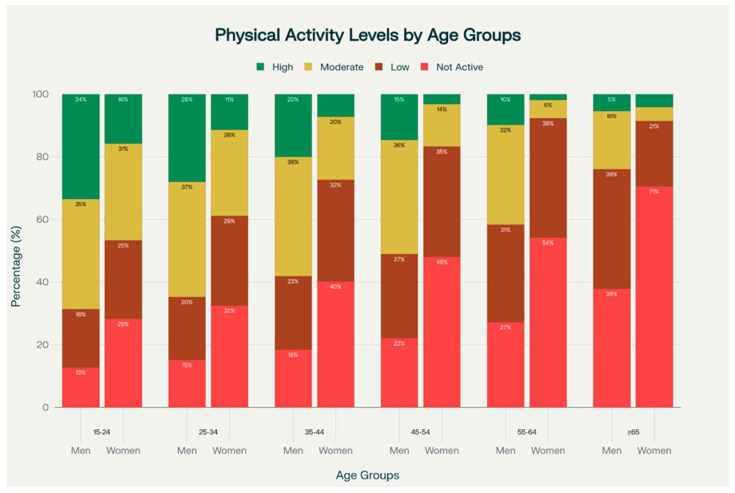
Age-Related Gradient in Physical Activity Levels by Sex (SHIS 2013). Stacked bar charts depict the prevalence of four physical activity categories—Not Active (<150 min/week MVPA), Low Activity (150–299 min/week MVPA), Moderate Activity (300–449 min/week MVPA), and High Activity (≥450 min/week MVPA)—across six age groups (15–24, 25–34, 35–44, 45–54, 55–64, and ≥65 years) for men (left bars) and women (right bars). Data from the 2013 Saudi Health Interview Survey indicate an apparent increase in physical inactivity with age, particularly among women, who experience a rise from 28% inactivity at 15–24 years to 71% at ≥65 years. Men’s inactivity increases from 13% to 38%. High activity levels decline significantly with age in both sexes, highlighting a notable gender disparity, particularly in those aged ≥ 55.

This results in a descriptive female-male difference of +32.7 percentage points (pp). A small minority of respondents reached the IPAQ “High” activity category within this age group (further details available in the SHIS tables) (See Figure 1; Table 1).

Sedentary behavior.

- General sitting ≥ 6 h/day (excludes sleep). Women: 42.7% (95% CI 34.7–50.7); Men: 32.0% (95% CI 26.3–37.7). Female–male difference: +10.7 pp.
- Television/computer ≥ 6 h/day. Women: 10.9% (95% CI 5.6–16.2); Men: 6.0% (95% CI 3.5–8.5). Female–male difference: +4.9 pp.

**Figure 2:**
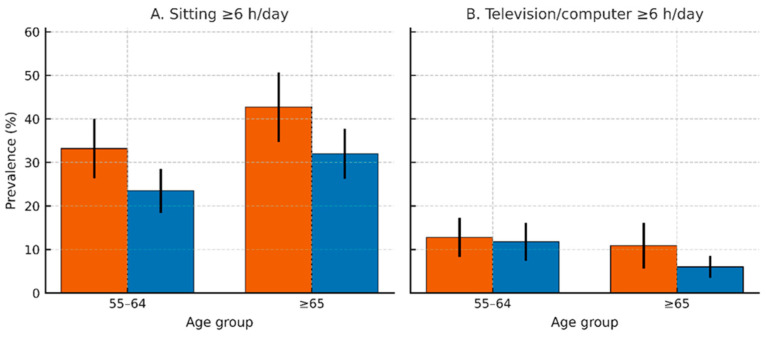
Prevalence of Sedentary Behaviors Among Adults Aged 55–64 and ≥65 Years, by Sex (SHIS 2013). Grouped bar charts with standard error bars illustrate two dimensions of sedentary behavior: (**A**) general sitting time (≥6 h/day, excluding sleep) and (**B**) screen-based sedentary time (≥6 h/day television or computer use), differentiated by age group (55–64 years vs. ≥65 years) and sex (men in blue, women in orange). Findings show that general sitting time increases with age in both sexes, with women reporting a higher prevalence (33.2% vs. 42.7% for women; 23.5% vs. 32.0% for men). Conversely, screen-based sedentary time slightly declines with age (women: 12.8% to 10.9%; men: 11.8% to 6.0%), particularly among men.

Prolonged general sitting was notably more common than television/computer ≥ 6 h/day, indicating that non-screen sitting dominates sedentary behavior in later life (Figure 2A,B; Table 1).

### 3.2. Descriptive Step-Change from Ages 55–64 to ≥65

The unweighted sample consisted of individuals aged 55–64 years, with 862 participants (women, n = 423; men, n = 439). We present absolute percentage-point (Δpp) differences (≥65 minus 55–64) within sex; CIs for Δ are not computed due to missing covariance.

Physical inactivity.
Women: 54.2% (95% CI 47.5–60.9) at 55–64 to 70.6% (95% CI 63.4–77.8) at ≥65; Δ = +16.4 pp.
Men: 27.2% (95% CI 22.0–32.4) to 37.9% (95% CI 32.4–43.4); Δ = +10.7 pp.
General sitting ≥ 6 h/day.
Women: 33.2% (95% CI 26.4–40.0) to 42.7% (95% CI 34.7–50.7); Δ = +9.5 pp.
Men: 23.5% (95% CI 18.4–28.6) to 32.0% (SE 2.93; 95% CI 26.3–37.7); Δ = +8.5 pp.
Television/computer ≥ 6 h/day.
Women: 12.8% (95% CI 8.3–17.3) to 10.9% (SE 2.69; 95% CI 5.6–16.2); Δ = −1.9 pp.
Men: 11.8% (95% CI 7.4–16.2) to 6.0% (95% CI 3.5–8.5); Δ = −5.8 pp.

**Figure 3:**
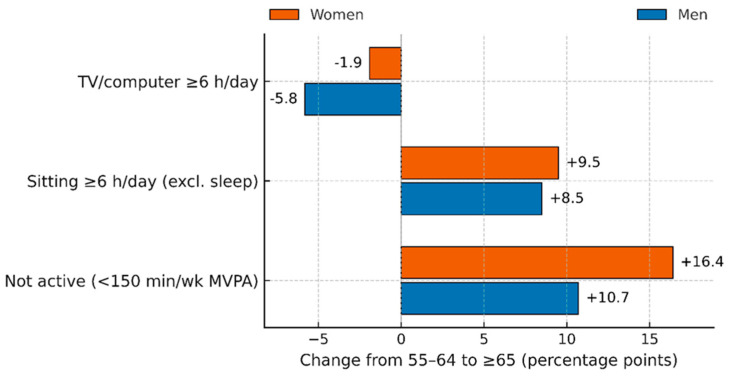
Magnitude of Behavioral Change at the Retirement Threshold: Step-Change from Ages 55–64 to ≥65 Years, by Sex (SHIS 2013). A horizontal bar chart illustrates the percentage point change in three behaviors—physical inactivity, general sitting time, and screen-based sedentary time—comparing late middle age (55–64 years) to older adulthood (≥65 years), by sex (women in orange, men in blue). At age 65, inactivity and sitting times increase significantly (women: +16.4 and +9.5 pp; men: +10.7 and +8.5 pp), while screen time slightly decreases (women: −1.9 pp; men: −5.8 pp).

Key pattern. Across both sexes, the increase in overall sedentary time with age is driven by non-screen sitting, while high television/computer time declines modestly (Figure 3; Table 2).

### 3.3. Summary

The transition from ages 55–64 to 65 years and beyond represents a descriptive inflection point in movement patterns. Both inactivity and general sitting increased substantially across both sexes at this age threshold, coinciding with the typical retirement transition in Saudi Arabia (retirement age ≥ 65 years).

Women experienced steeper increases in inactivity (+16.4 percentage points vs. +10.7 percentage points in men) and general sitting (+9.5 percentage points vs. +8.5 percentage points in men), underscoring the greater behavioral vulnerability of the female cohort to the structural changes associated with retirement. Screen-based sedentary time declined slightly with age (+1.9 percentage points in women; +5.8 percentage points in men), a pattern consistent with generational differences in technology use rather than age-related increases in screen engagement.

## 4. Discussion

Three observations are central and together define the contribution of this work. First, there is a pronounced sex disparity in physical inactivity at ages ≥ 65 years, quantified as a 32.7 percentage-point difference (women 70.6% vs. men 37.9%). Second, a descriptive step-change occurs at age ≥ 65, where inactivity increases by 16.4 percentage points in women and 10.7 percentage points in men, and prolonged sitting rises by 9.5 and 8.5 percentage points, respectively, when compared with ages 55–64. Third, age-related increases in sedentary time are primarily driven by non-screen sitting (e.g., passive rest, social sitting, transportation), whereas television/computer time declines with age, particularly among men.

### 4.1. Principal Findings

Taken together, the results depict a dual burden in later life: high levels of physical inactivity coupled with substantial sedentary behavior, with the burden consistently higher among women aged ≥ 65 years. The transition from 55–64 to ≥65 is accompanied by an unambiguous descriptive shift toward more inactivity and more time spent sitting in both sexes. In contrast, screen-based sedentary time decreases modestly, especially among men. These contrasts indicate that interventions should prioritize increasing overall activity while reducing non-screen sitting rather than focusing narrowly on screen time.

### 4.2. Explaining the Sex Disparity

The magnitude of the female–male gap aligns with long-standing structural and sociocultural barriers that have constrained women’s opportunities for active living, including limited access to women-only facilities, safe proximate spaces, and convenient transport [10,11]. Life course influences are also plausible: the cohorts observed here formed adult habits largely before recent policy reforms, and norms emphasizing domestic roles over discretionary health behaviors have likely shaped choices across decades [28,29]. In this context, gains from contemporary initiatives may accrue more quickly to younger cohorts, with slower penetration among the oldest age groups. A gender-responsive strategy remains necessary, including neighborhood and mosque-based walking groups, women-only exercise options, home-based outreach when needed, and social prescribing that links older women to culturally congruent, low-barrier resources [28,29,30].

### 4.3. The Retirement Transition as a Clinical and Policy Opportunity

Retirement reorganizes daily time use, removes work-related incidental movement, and introduces larger blocks of unstructured time, conditions that can promote or hinder health behavior change [13,14,31,32]. Evidence supports the premise that this period functions as a teachable moment in which brief counseling and structured supports are particularly effective [33,34]. A pragmatic sequence is feasible. Pre-retirement counseling (approximately ages 58–62) embedded in routine care can initiate planning for activity and sitting reduction; newly retired adults (approximately ages 63–70) can be engaged through community walking groups, supervised exercise classes, and social clubs; and primary care can normalize screening for activity and sitting, provide brief advice, and make referrals to local resources. Group-based aerobic and resistance training delivered in community health centers, rather than specialized facilities, can reduce access barriers and improve adherence [35,36].

### 4.4. Frailty, Sarcopenia, and Integrated Nutritional Considerations

The observed profiles have implications beyond mobility. Physical inactivity and prolonged sitting are closely associated with frailty and sarcopenia, which elevate risks of falls, hospitalization, and mortality [37,38]. Converging evidence also indicates that sedentary, inactive older adults often present with unfavorable nutritional status and oxidative-stress profiles, suggesting a pathway that amplifies biological vulnerability [21,22]. These patterns support an integrated clinical response in which routine assessment of activity and daily sitting is coupled with nutrition evaluation focused on protein adequacy and relevant micronutrients, and where referral to progressive resistance training of sufficient duration (≥8 weeks) is used to improve strength, gait speed, and functional capacity [35,36]. In parallel, the broader public health framework for activity and sedentary behavior provides a coherent basis for clinical messaging and program goals [7].

### 4.5. Strengths and Limitations

The analysis has several strengths. It is nationally representative across all 13 regions, provides sex-stratified estimates with 95% confidence intervals, and examines inactivity alongside two sedentary indicators, enabling consistent interpretation. It adheres transparently to a descriptive analytic framework that utilizes design-weighted published survey tables and offers a 2013 baseline against which subsequent changes can be evaluated. Important limitations qualify interpretation. All outcomes are self-reported, and questionnaire-based estimates of sedentary time typically differ from device-based measures. The 2013 time frame predates major policy and environmental changes; estimates should be read as a baseline rather than current prevalence. The cross-sectional design yields age-band contrasts that are descriptive, not longitudinal changes, and causal inference is not warranted. Reliance on aggregated tables rather than microdata precludes multivariable adjustment for potential confounders (e.g., socioeconomic status, comorbidity, marital status). Because covariance between age bands is not available in the public tables, confidence intervals for differences were not computed, and step-changes are presented descriptively. Finally, the television/computer measure reflects the 2013 context and does not capture the later proliferation of mobile devices.

### 4.6. Clinical and Policy Implications and Future Directions

A practical clinical pathway can be integrated into routine geriatric care without significant resource demands. Screening for activity and daily sitting can be incorporated into standard assessments. Brief counseling can emphasize the independent benefits of increasing physical activity and reducing sedentary time, as well as setting realistic and achievable goals. Additionally, social prescribing can connect patients to nearby community resources and peer-support networks. Approaches should explicitly address the barriers faced by older women, including women-only sessions, transport solutions, and culturally congruent options that respect family roles and preferences. In parallel, nutrition assessment should be included when sedentary behavior and inactivity are prominent, given the links between movement profiles, micronutrient status, and oxidative stress [21,22]. At the policy level, progress depends on having safe, shaded, and proximate walking infrastructure; reliable transport links to community venues; workplace policies that support pre-retirement planning for physical activity; and the integration of screening, counseling, and referral into primary care pathways. Looking ahead, research priorities include longitudinal cohorts that follow adults across the retirement transition, device-based measurement to validate self-reports, analyses using individual-level microdata to allow multivariable adjustment, qualitative studies that elucidate older women’s lived experiences and specific barriers, and implementation research that evaluates feasibility, reach, and effectiveness in routine practice.

## 5. Conclusions

Older Saudi adults, particularly women, face a significant dual challenge of physical inactivity and prolonged sedentary behavior, both of which escalate notably after the age of 65, coinciding with the retirement transition. This shift underscores the urgent need to promote active aging through targeted interventions. In alignment with the goals of Saudi Vision 2030, which prioritizes improving the quality of life and fostering a vibrant society, national strategies should focus on increasing physical activity and reducing non-screen sitting time among older populations. Effective solutions must be gender responsive, culturally sensitive, and accessible, such as women-only programs, transportation support, and community-based initiatives that cater to women. The retirement phase presents a strategic window for engagement, where even modest, sustained improvements, especially when paired with progressive resistance training and behavioral support, can significantly enhance strength, mobility, and overall well-being. This study provides a foundational national baseline to inform future health policies and community programs that support healthy aging in Saudi Arabia.

## Figures and Tables

**Table 1:** Prevalence of Physical Inactivity and Sedentary Behaviors Among Adults Aged ≥ 65 Years by Sex (SHIS 2013, Design-Weighted Estimates with Standard Errors and 95% Confidence Intervals).

Behavioral Outcome	Men ≥ 65 Years, % (n = 517)	SE	95% CI	Women ≥ 65 Years, % (n = 358)	SE	95% CI	Female–Male Difference (pp)
Not Active (<150 min/week MVPA)	37.9	2.82	32.3–43.5	70.6	3.69	63.4–77.8	+32.7
Sitting ≥ 6 h/day	32.0	2.93	26.3–37.7	42.7	4.07	34.7–50.7	+10.7
TV/Computer ≥ 6 h/day	6.0	1.28	3.5–8.5	10.9	2.69	5.7–16.1	+4.9

Percentages are design-weighted prevalence estimates; unweighted sample sizes (n) shown per sex–age group; SEs and 95% CIs calculated from published SE values. Abbreviations: pp, percentage points; SE, standard error; MVPA, moderate-to-vigorous physical activity; CI, confidence interval.

**Table 2:** Descriptive Step-Change in Physical Inactivity and Sedentary Behaviors from Ages 55–64 to ≥65 Years, by Sex.

Behavioral Outcome	Women 55–64, % (n = 423)	SE	95% CI	Women ≥ 65, % (n = 358)	SE	95% CI	Step-Change (pp)	Men 55–64, % (n = 439)	SE	95% CI	Men ≥ 65, % (n = 517)	SE	95% CI	Step-Change (pp)
Not Active (<150 min/week)	54.2	3.44	47.6–60.8	70.6	3.69	63.4–77.8	+16.4	27.2	2.63	21.9–32.5	37.9	2.82	32.3–43.5	+10.7
Sitting ≥ 6 h/day	33.2	3.48	26.4–40.0	42.7	4.07	34.7–50.7	+9.5	23.5	2.58	18.5–28.5	32.0	2.93	26.3–37.7	+8.5
TV/Computer ≥ 6 h/day	12.8	2.31	8.3–17.3	10.9	2.69	5.7–16.1	−1.9	11.8	2.23	7.5–16.1	6.0	1.28	3.5–8.5	−5.8

Percentages are design-weighted prevalence estimates; unweighted sample sizes (n) shown per sex–age group: SEs and 95% CIs calculated from published SE values. Step-changes reported as absolute percentage point differences without CIs due to unavailable covariance matrices for contrasts. Abbreviations: pp, percentage points; SE, standard error; CI, confidence interval.

## Data Availability

All data analyzed in this study were extracted from the publicly available Saudi Health Interview Survey (SHIS) 2013 report, which can be accessed at: (https://www.healthdata.org/sites/default/files/files/Projects/KSA/Saudi-Health-Interview-Survey-Results.pdf, accessed on 25 September 2025).

## Abbreviations

The following abbreviations are used in this manuscript:

Abbreviation	Definition
CAPI	Computer-Assisted Personal Interviewing
CI	Confidence Interval
GHDx	Global Health Data Exchange
IHME	Institute for Health Metrics and Evaluation
IPAQ	International Physical Activity Questionnaire
METs	Metabolic Equivalents
MOH	Ministry of Health (Saudi Arabia)
MVPA	Moderate-to-Vigorous Physical Activity
NCDs	Noncommunicable Diseases
pp	Percentage Points
PSUs	Primary Sampling Units
SBRN	Sedentary Behavior Research Network
SE	Standard Error
SHIS	Saudi Health Interview Survey
STROBE	Strengthening the Reporting of Observational Studies in Epidemiology
WHO	World Health Organization

## References

[1] Lee I.M., Shiroma E.J., Lobelo F., Puska P., Blair S.N., Katzmarzyk P.T. (2012). Effect of physical inactivity on major non-communicable diseases worldwide: An analysis of burden of disease and life expectancy. Lancet.

[2] Biswas A., Oh P.I., Faulkner G.E., Bajaj R.R., Silver M.A., Mitchell M.S., Alter D.A. (2015). Sedentary time and its association with risk for disease incidence, mortality, and hospitalization in adults: A systematic review and meta-analysis. Ann. Intern. Med..

[3] Thornton J.S., Morley N. (2025). Move more, age well: Prescribing physical activity for older adults. Can. Med Assoc. J..

[4] Ekelund U., Tarp J., Steene-Johannessen J., Hansen B.H., Jefferis B., Fagerland M.W., Whincup P., Diaz K.M., Hooker S.P., Chernofsky A. (2019). Dose-response associations between accelerometry measured physical activity and sedentary time and all cause mortality: Systematic review and harmonised meta-analysis. BMJ.

[5] Watts E.L., Saint-Maurice P.F., Dempsey P.C., Hamer M., Stamatakis E., Matthews C.E. (2022). Association of leisure time physical activity types and risks of all-cause, cardiovascular, and cancer mortality among older adults. JAMA Netw. Open.

[6] Hansen B.H., Eidem A., Koster A., Penninx B.W., Pedersen K.K., Sagelv E.H., Hopstock L.A., Egerton T., Aspvik N.P., Jacobsen B.K. (2024). Physical activity and all-cause mortality by age in 4 multinational megacohorts. JAMA Netw. Open.

[7] Bull F.C., Al-Ansari S.S., Biddle S., Borodulin K., Buman M.P., Cardon G., Carty C., Chaput J.P., Chastin S., Chou R. (2020). World Health Organization 2020 guidelines on physical activity and sedentary behaviour. Br. J. Sports Med..

[8] Guthold R., Stevens G.A., Riley L.M., Bull F.C. (2018). Worldwide trends in insufficient physical activity from 2001 to 2016: A pooled analysis of 358 population-based surveys with 1·9 million participants. Lancet Glob. Health.

[9] Alqahtani B.A., Alenazi A.M., Alshehri M.M., Osailan A.M., Alsubaie S.F., Alqahtani M.A. (2021). Prevalence of frailty and associated factors among Saudi community-dwelling older adults: A cross-sectional study. BMC Geriatr..

[10] Alasmari H.D., Alshehri M.A. (2018). Physical inactivity in Saudi Arabia revisited: A systematic review of inactivity prevalence and perceived barriers to active living. Int. J. Health Sci..

[11] Abdelhay O., Altamimi M., Abdelhay Q., Manajrah M., Tourkmani A.M., Altamimi M., Altamimi T. (2025). Perceived barriers to physical activity and their predictors among adults in the Central Region in Saudi Arabia: Gender differences and cultural aspects. PLoS ONE.

[12] Alqahtani S.A., AlAhmed R., Hamza M.M., Alessy S.A., Alqunaibet A., AlGhammas A., Watkins D., Msemburi W., Alkhattabi F., Pickersgill S. (2024). Health and economic burden of insufficient physical activity in Saudi Arabia. PLoS ONE.

[13] McDonald S., O’Brien N., White M., Sniehotta F.F. (2015). Changes in physical activity during the retirement transition: A theory-based, qualitative interview study. Int. J. Behav. Nutr. Phys. Act..

[14] Barnett I., van Sluijs E.M., Ogilvie D. (2012). Physical activity and transitioning to retirement: A systematic review. Am. J. Prev. Med..

[15] Kingdom of Saudi Arabia Saudi Vision 2030. https://www.vision2030.gov.sa/.

[16] Owen N., Healy G.N., Matthews C.E., Dunstan D.W. (2010). Too much sitting: The population health science of sedentary behavior. Exerc. Sport Sci. Rev..

[17] Tremblay M.S., Colley R.C., Saunders T.J., Healy G.N., Owen N. (2010). Physiological and health implications of a sedentary lifestyle. Appl. Physiol. Nutr. Metab..

[18] Sedentary Behaviour Research Network (2012). Letter to the editor: Standardized use of the terms “sedentary” and “sedentary behaviours”. Appl. Physiol. Nutr. Metab..

[19] Owen N., Sparling P.B., Healy G.N., Dunstan D.W., Matthews C.E. (2010). Sedentary behavior: Emerging evidence for a new health risk. Mayo Clin. Proc..

[20] Ekelund U., Steene-Johannessen J., Brown W.J., Fagerland M.W., Owen N., Powell K.E., Bauman A., Lee I.M. (2016). Does physical activity attenuate, or even eliminate, the detrimental association of sitting time with mortality? A harmonised meta-analysis of data from more than 1 million men and women. Lancet.

[21] Mądra-Gackowska K., Szewczyk-Golec K., Gackowski M., Hołyńska-Iwan I., Parzych D., Czuczejko J., Graczyk M., Husejko J., Jabłoński T., Kędziora-Kornatowska K. (2025). Selected Biochemical, Hematological, and Immunological Blood Parameters for the Identification of Malnutrition in Polish Senile Inpatients: A Cross-Sectional Study. J. Clin. Med..

[22] Mądra-Gackowska K., Szewczyk-Golec K., Gackowski M., Woźniak A., Kędziora-Kornatowska K. (2023). Evaluation of Selected Parameters of Oxidative Stress and Adipokine Levels in Hospitalized Older Patients with Diverse Nutritional Status. Antioxidants.

[23] Saudi Ministry of Health, Institute for Health Metrics and Evaluation (2013). Saudi Health Interview Survey Results.

[24] Tremblay M.S., Aubert S., Barnes J.D., Saunders T.J., Carson V., Latimer-Cheung A.E., Chastin S.F.M., Altenburg T.M., Chinapaw M.J.M., SBRN Terminology Consensus Project Participants (2017). Sedentary Behavior Research Network (SBRN)—Terminology Consensus Project process and outcome. Int. J. Behav. Nutr. Phys. Act..

[25] Rosenberg D.E., Bull F.C., Marshall A.L., Sallis J.F., Bauman A.E. (2008). Assessment of sedentary behavior with the International Physical Activity Questionnaire. J. Phys. Act. Health.

[26] Healy G.N., Clark B.K., Winkler E.A., Gardiner P.A., Brown W.J., Matthews C.E. (2011). Measurement of adults’ sedentary time in population-based studies. Am. J. Prev. Med..

[27] von Elm E., Altman D.G., Egger M., Pocock S.J., Gøtzsche P.C., Vandenbroucke J.P., STROBE Initiative (2014). The Strengthening the Reporting of Observational Studies in Epidemiology (STROBE) Statement: Guidelines for reporting observational studies. Int. J. Surg..

[28] Baert V., Gorus E., Mets T., Geerts C., Bautmans I. (2011). Motivators and barriers for physical activity in the oldest old: A systematic review. Ageing Res. Rev..

[29] Costello E., Kafchinski M., Vrazel J., Sullivan P. (2011). Motivators, barriers, and beliefs regarding physical activity in an older adult population. J. Geriatr. Phys. Ther..

[30] Alsubaie A.S. (2015). Lack of facilities rather than sociocultural factors as the primary barrier to physical activity among female Saudi university students. Int. J. Womens Health.

[31] Stenholm S., Pulakka A., Kawachi I., Oksanen T., Halonen J.I., Aalto V., Kivimäki M., Vahtera J. (2016). Changes in physical activity during transition to retirement: A cohort study. Int. J. Behav. Nutr. Phys. Act..

[32] Lawton R., Conner M., McEachan R. (2009). Desire or reason: Predicting health behaviors from affective and cognitive attitudes. Health Psychol..

[33] Michie S., Abraham C., Whittington C., McAteer J., Gupta S. (2009). Effective techniques in healthy eating and physical activity interventions: A meta-regression. Health Psychol..

[34] Serra-Prat M., Sist X., Saiz A., Arnau A., Roquet M., Yebenes J.C., Papiol M. (2017). Effectiveness of an intervention to prevent frailty in pre-frail community-dwelling older people consulting in primary care: A randomised controlled trial. Age Ageing.

[35] de Labra C., Guimaraes-Pinheiro C., Maseda A., Lorenzo T., Millán-Calenti J.C. (2015). Effects of physical exercise interventions in frail older adults: A systematic review of randomized controlled trials. BMC Geriatr..

[36] Zhao Y., Zhang Y., Hao Q., Ge M., Dong B. (2023). The association between sedentary behaviour and sarcopenia in older adults: A systematic review and meta-analysis. BMC Geriatr..

[37] Cruz-Jentoft A.J., Bahat G., Bauer J., Boirie Y., Bruyère O., Cederholm T., Cooper C., Landi F., Rolland Y., Sayer A.A. (2019). Sarcopenia: Revised European consensus on definition and diagnosis. Age Ageing.

[38] Bindawas S.M. (2023). The Changing Incidence and Prevalence of Falls and Its Disability Burden Among the Geriatric Population in Saudi Arabia from 1990 to 2019: A Longitudinal Analysis Using Global Burden of Disease Study Data. Cureus.

